# Magnetosheath Jets Over Solar Cycle 24: An Empirical Model

**DOI:** 10.1029/2023JA031493

**Published:** 2023-08-15

**Authors:** Laura Vuorinen, Adrian T. LaMoury, Heli Hietala, Florian Koller

**Affiliations:** ^1^ Department of Physics and Astronomy University of Turku Turku Finland; ^2^ Blackett Laboratory Imperial College London London UK; ^3^ Department of Physics and Astronomy Queen Mary University of London London UK; ^4^ Institute of Physics University of Graz Graz Austria

## Abstract

Time History of Events and Macroscale Interactions during Substorms (THEMIS) spacecraft have been sampling the subsolar magnetosheath since the first dayside science phase in 2008, and we finally have observations over a solar cycle. However, we show that the solar wind coverage during these magnetosheath intervals is not always consistent with the solar wind conditions throughout the same year. This has implications for studying phenomena whose occurrence depends strongly on solar wind parameters. We demonstrate this with magnetosheath jets—flows of enhanced earthward dynamic pressure in the magnetosheath. Jets emerge from the bow shock, and some of them can go on and collide into the magnetopause. Their occurrence is highly linked to solar wind conditions, particularly the orientation of the interplanetary magnetic field, as jets are mostly observed downstream of the quasi‐parallel shock. We study the yearly occurrence rates of jets recorded by THEMIS over solar cycle 24 (2008–2019) and find that they are biased due to differences in spacecraft orbits and uneven sampling of solar wind conditions during the different years. Thus, we instead use the THEMIS observations and their corresponding solar wind conditions to develop a model of how jet occurrence varies as a function of solar wind conditions. We then use OMNI data of the whole solar cycle to estimate the unbiased yearly jet occurrence rates. For comparison, we also estimate jet occurrence rates during solar cycle 23 (1996–2008). Our results suggest that there is no strong solar cycle dependency in jet formation.

## Key Points

- Observed jet occurrence rates can be biased due to spacecraft orbits and uneven solar wind sampling
- We created a statistical model of jet occurrence using interplanetary magnetic field cone angle, magnitude, solar wind speed, and density
- There is no strong solar cycle dependency in jet occurrence, but there may be a ∼10%–20% decrease around solar maximum

## 1 Introduction

Magnetosheath jets are localized enhancements of dynamic pressure downstream of the Earth's bow shock (see the review by Plaschke et al., 2018, and the references therein). These jets emerge from the shock and they propagate toward the Earth with some of them eventually impacting the magnetopause. The occurrence of jets is highly dependent on solar wind (SW) conditions, most importantly the orientation of the interplanetary magnetic field (IMF). When the angle between the Sun‐Earth line and the IMF (the IMF cone angle) is small, the subsolar magnetosheath is downstream of a quasi‐parallel shock, and jets occur most frequently (Archer & Horbury, 2013; Plaschke et al., 2013; Vuorinen et al., 2019). Therefore, suggested jet formation mechanisms are mostly related to the quasi‐parallel shock and the foreshock region upstream of it: foreshock structures such as short large‐amplitude magnetic structures (SLAMS; Schwartz, 1991) entering the magnetosheath (Karlsson et al., 2015; Palmroth et al., 2018; Suni et al., 2021), solar wind traveling through ripples on the bow shock (Hietala et al., 2009; Hietala & Plaschke, 2013), and solar wind being trapped into downstream during the shock reformation process (Raptis et al., 2022).

The growing number of Time History of Events and Macroscale Interactions during Substorms (THEMIS) spacecraft (Angelopoulos, 2008) observations in the subsolar magnetosheath have made possible extensive statistical studies, which have advanced our understanding of solar wind conditions affecting jet occurrence. Vuorinen et al. (2019) found that jet occurrence is 9 times higher downstream of the quasi‐parallel shock than downstream of the quasi‐perpendicular shock. LaMoury et al. (2021) studied separately jets observed close to the bow shock and those close to the magnetopause to disentangle the solar wind influence on jet formation and propagation to the magnetopause. They found that, in addition to the IMF cone angle, jet formation seems to be increased during low IMF magnitude *B*, low SW density *n*, high plasma *β*, and high Alfvén Mach number *M*_A_. Koller et al. (2022) studied jets during large‐scale solar wind structures, and found an increase in jet occurrence during stream‐interaction regions/co‐rotating interaction regions (SIRs/CIRs) and high‐speed streams (HSSs), but a decrease during magnetic ejecta and sheath regions of coronal mass ejections (CMEs). Koller et al. (2023) continued this investigation and found that high IMF cone angle and high Alfvén Mach conditions are unfavorable for jet occurrence, which makes jet occurrence rates during CMEs lower. Similarly, they found that conditions typical for HSSs (low IMF cone angle, low density, low IMF strength) are very favorable for jet generation. As the frequency of these structures and the characteristics of the solar wind vary across a solar cycle, a natural question arises: how does the formation of magnetosheath jets vary during the solar cycle? We now have THEMIS measurements from the subsolar magnetosheath from the years 2008–2020 that span over the solar cycle 24. In this paper we aim to answer this question by studying the yearly jet occurrence rates close to the bow shock.

Comparing yearly jet observation rates can be challenging. Jets are much more frequently observed close to the bow shock, so the number of observed jets varies depending on the spacecraft's location in the magnetosheath. The apogees of THEMIS spacecraft change throughout the years. We can control for this bias by focusing only on jet observations close to the bow shock. However, when the spacecraft apogees are low, the spacecraft are close to the bow shock only during such solar wind conditions when the shock has moved substantially earthward. This leads to a bias in solar wind condition coverage and consequently in the jet occurrence rates. To obtain unbiased jet occurrence rates for each year, we build a statistical model of jet occurrence as a function of solar wind conditions using the THEMIS measurements from 2008 to 2020 and their corresponding OMNI measurements. To reconstruct unbiased yearly jet occurrence rates, we then input all OMNI solar wind observations throughout the solar cycle into the model. This reconstruction shows that there is no strong solar cycle variation in jet occurrence, in contrast to the biased THEMIS observations which show a large decrease in jet occurrence during the solar maximum. More generally, our results highlight the need for careful normalization when analyzing statistical data sets of phenomena that are dependent on location in the magnetosheath and on solar wind conditions.

This paper is organized as follows. First, we introduce the THEMIS data set used in this study. Second, we show how the solar wind conditions vary during solar cycle 24 and during THEMIS dayside coverage and present the biased yearly jet occurrence rates observed by THEMIS spacecraft. We then describe how we build the statistical model to account for these biases. Following this, we create the model, show how it performs and finally present estimations of the unbiased jet occurrence rates across the solar cycle 24. For comparison, we also show the estimations for the previous cycle 23.

## 2 Observations

### 2.1 Data Sets

**Figure 1:**
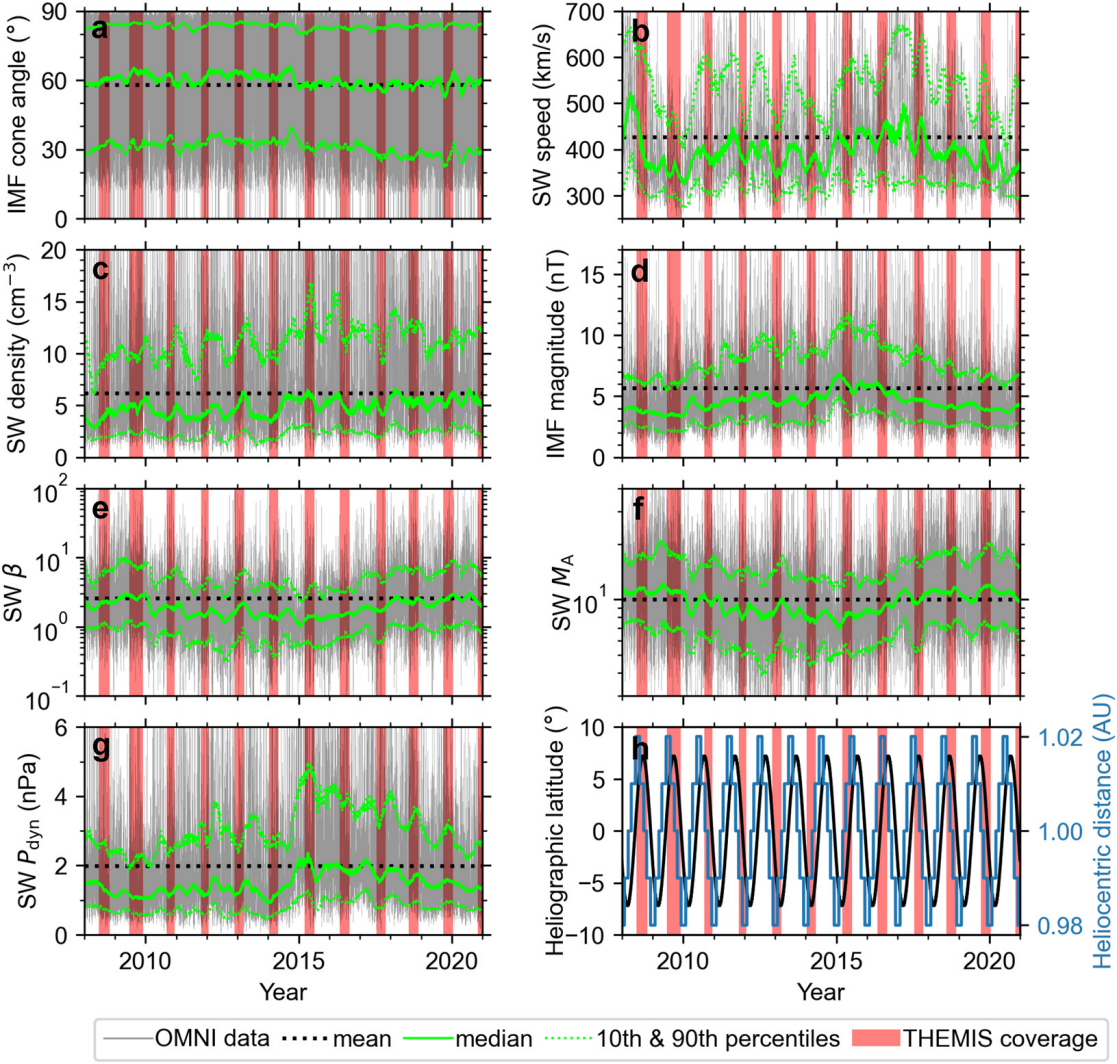
OMNI observations for the years 2008–2020 (solar cycle 24; from December 2008 until December 2019): (a) IMF cone angle, (b) solar wind speed, (c) density, (d) IMF magnitude, (e) *β*, (f) Alfvén Mach number, and (g) dynamic pressure. Gray lines show the hourly observations, red highlights the subsolar magnetosheath intervals of THEMIS, and the black dotted line shows the means of the quantities. The green solid line shows the running 90‐day median, and the green dotted lines show the running 90‐day 10th and 90th percentiles. Panel (h) shows Earth's heliographic latitude (black) and heliocentric distance (blue).

We use a magnetosheath jet data set first presented by Koller et al. (2022) following the criteria introduced by Plaschke et al. (2013). It is based on THEMIS on‐board moment data from the subsolar magnetosheath. THEMIS orbits undergo a yearly drift around the Earth due to the motion of the Earth around the Sun (Angelopoulos, 2008) such that their apogees will sometimes be at the flanks or in the tail so that they will not cross into the subsolar magnetosheath at all. This means that THEMIS spacecraft will inevitably be sampling that region only a fraction of the year or the solar cycle (around 1/4 of the time as indicated by the red highlights in Figure 1). THEMIS spacecraft were required to be within a 7–18 *R*_E_ geocentric distance and inside a 30° Sun‐facing cone with the Sun‐Earth line as its axis. The solar wind and IMF conditions are from the 1‐min high‐resolution OMNI data set (King & Papitashvili, 2005), which we average over the five preceding minutes for a given magnetosheath observation. To ensure that the spacecraft were in fact in the magnetosheath, the THEMIS density measurements had to be over twice the density observed in the solar wind. Additionally, the energy flux of 1 keV ions had to be larger than that of 10 keV ions to exclude inner magnetospheric observations. Only magnetosheath intervals longer than 2 min were included.

The main criterion of Plaschke et al. (2013) for magnetosheath jets is that the earthward (−*X*_GSE_) dynamic pressure has to exceed 50% of the corresponding solar wind dynamic pressure. The whole jet interval around it is then defined as the period during which the earthward dynamic pressure stays above 25% of the solar wind dynamic pressure. We follow the notation of Plaschke et al. (2013) and denote the time of the maximum dynamic pressure ratio between a jet and the upstream solar wind as *t*_0_. In this study, each jet is represented by the observation at its *t*_0_. At some point in the 1‐min intervals before and after the jet interval, *V*_*X*_ also has to surpass *V*_*X*_(*t*_0_)/2. This velocity criterion excludes density enhancements in steady magnetosheath flow. We note that the conclusions of this study remain while using a separate jet list introduced by Koller et al. (2022), which applies a local magnetosheath criterion for earthward dynamic pressure enhancements and does not include this velocity criterion. The list includes earthward dynamic pressure enhancements larger than three times the local 20‐min running average in the magnetosheath, and it can also be found online (Koller et al., 2021).

Recently, some concern has raised concerning the calibration of THEMIS E on‐board moments during the later years of the mission, as these density and velocity measurements can deviate from ground moment measurements. THEMIS E observes more jets than THEMIS A and D (Koller et al., 2022). The results shown in this manuscript have been obtained using all data, but to ensure that this does not change our conclusions, we also reproduced the results while conservatively neglecting all THEMIS E data.

### 2.2 Solar Wind Observations During THEMIS Dayside Coverage

Figure 1 presents OMNI solar wind observations for the years 2008–2020 (panels a–g) and Earth's heliographic latitude and heliocentric distance (panel h), spanning over the solar cycle 24 which lasted from December 2008 to December 2019. Many of the parameters exhibit variations across the solar cycle: IMF magnitude is smaller during solar minimum and *β* and *M*_A_ are smaller during solar maximum. The sharp dynamic pressure increase observed here during 2014 (Figure 1g; close to solar maximum) is similar to a global phenomenon (across all heliolatitudes) observed during other solar cycles (J. Richardson & Wang, 1999). We presume this may related to stream interaction regions being most prevalent during the declining phase of the solar cycle (I. G. Richardson & Cane, 2012). We also see periodicity on a scale of around a year, perhaps influenced by the varying heliographic latitude and heliocentric distance (Figure 1h). The time periods filled with red are the THEMIS observation intervals in the subsolar magnetosheath (as determined by the criteria of Plaschke et al., 2013). These intervals can coincide with the periodicity of the solar wind, which may lead to unrepresentative distributions of solar wind quantities for a given year. The most interesting parameter, from the perspective of magnetosheath jets, is the IMF cone angle. In Figure 1a, we can see that there are no substantial differences in its distribution throughout the solar cycle. This agrees with results reported by Samsonov et al. (2019), who investigated long‐term variations in OMNI solar wind parameters relevant for solar wind‐magnetosphere interactions over multiple solar cycles.

Jets are most frequently observed close to the bow shock, and their occurrence rates decrease substantially (by a factor of ∼6) from the bow shock to the magnetopause (LaMoury et al., 2021; Plaschke et al., 2013). Thus, the spacecraft's relative position in the magnetosheath affects how many jets it observes. This relative position is determined by two factors: the orbit of the spacecraft and the locations of the bow shock and the magnetopause. The apogees, where the spacecraft spent most of their time, of THEMIS orbits vary throughout the years and solar wind conditions control the locations of the boundaries. We can estimate the relative radial distance, or the fractional distance (e.g., Dimmock & Nykyri, 2013), *F* (*F* = 0 at magnetopause and *F* = 1 at bow shock) in the magnetosheath by using the Shue et al. (1998) magnetopause model and the Merka et al. (2005) bow shock model:

(1) $$F=\left(r-r_{\text{MP}}\right)/\left(r_{\text{BS}}-r_{\text{MP}}\right).$$

here *r* is the geocentric distance of the spacecraft and *r*_BS_ and *r*_MP_ are the geocentric distances of the model bow shock and magnetopause along that same line. We consider observations with *F* ∈ [0.5, 1.1] to be close to the bow shock. This selection yields 3,400 hr of magnetosheath observations and 9,566 jets. Note that due to uncertainties in the model boundaries, while the spacecraft are truly in the magnetosheath, they can be outside of the model magnetosheath.

**Figure 2:**
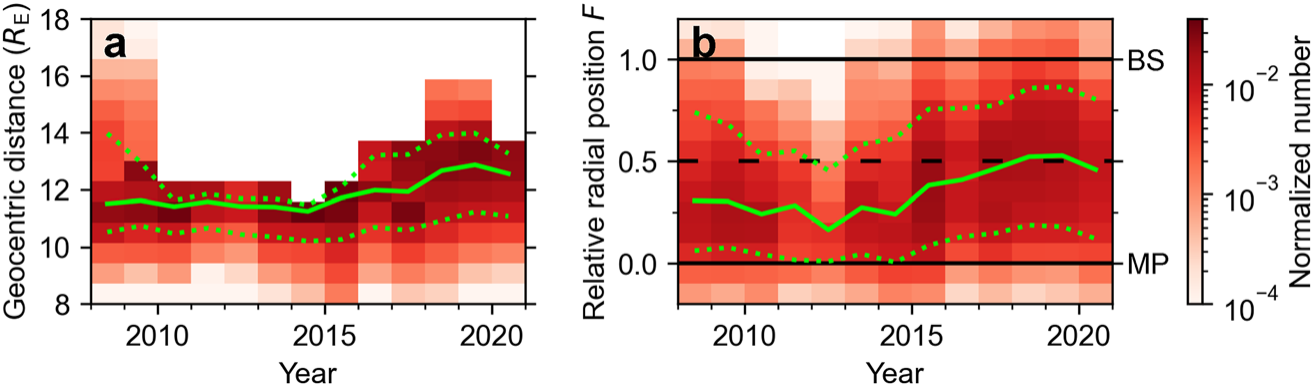
Distributions of (a) geocentric distances and (b) relative radial positions (between model bow shock at *F* = 1 and model magnetopause at *F* = 0) of THEMIS spacecraft during subsolar magnetosheath observations (i.e., the time spent at different locations) in years 2008–2020. The green solid line shows the yearly median, and the green dotted lines show the yearly 10th and 90th percentiles.

In Figure 2, we show the geocentric distances of the THEMIS spacecraft and the relative radial positions *F* during the subsolar magnetosheath observations. We can see that the apogees of THEMIS spacecraft were lower in 2010–2014. THEMIS B and C orbited the Earth with high apogees during 2008–2009, but they have been since then moved to orbit the Moon as the ARTEMIS probes (Angelopoulos, 2011). Due to the orbits, there are relatively fewer observations (only around 10% annually, see Figure 2b) close to the bow shock around 2010–2014. This may affect annual THEMIS observations of various bow shock related phenomena. The apogees were raised from 2015 onwards.

**Figure 3:**
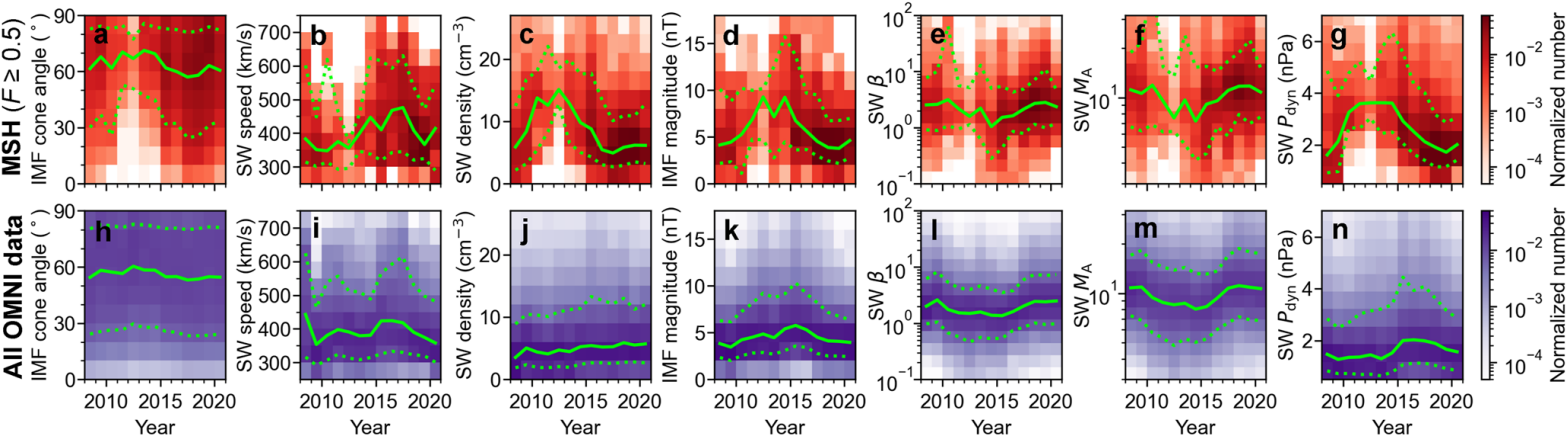
(a–g) OMNI solar wind observations linked to THEMIS subsolar magnetosheath observations close to the bow shock (*F* ∈ [0.5, 1.1]) from years 2008–2020 (solar cycle 24; from December 2008 until December 2019). (h–n) All OMNI observations from years 2008–2020. (a, h) IMF cone angle, (b, i) solar wind speed, (c, j) density, (d, k) IMF magnitude, (e, l) *β*, (f,m) Alfvén Mach number, and (g, n) dynamic pressure. The yearly medians are shown in solid green line and the 10th and 90th percentiles are shown in dotted green lines.

We focus on jets in the outer half of the magnetosheath (*F* ∈ [0.5, 1.1]). This allows us to control for the expected bias in jet occurrence due to the orbital variation. Importantly, LaMoury et al. (2021) showed that jet formation at the bow shock and the jet propagation to the magnetopause are influenced differently by the upstream solar wind conditions. Thus, focusing on the region close to the bow shock also allows us to concentrate on jet formation. Figure 2b showed that this region was not evenly covered by the spacecraft orbits. In Figure 3, we show the distributions of OMNI measurements during these THEMIS magnetosheath measurements (panels a–g, in red). For comparison, we also show all OMNI measurements during these years (panels h–n, in purple). We notice that OMNI measurements for the THEMIS magnetosheath intervals do not share the same distribution as all OMNI measurements during the period 2008–2020. In particular, there are large differences between these two distributions during years 2011–2014. This is most visible for IMF cone angle, solar wind density, IMF magnitude, and dynamic pressure. The IMF cone angle being the most significant parameter controlling jet occurrence, THEMIS observed less favorable conditions for jet occurrence. This means that there will be a bias also in the annual jet occurrence rates during these years. There are several possible reasons for these less favorable conditions for jet occurrence. First, as the apogees were lower, the spacecraft were close to the bow shock only during conditions when the magnetosphere was compressed and the bow shock was pushed earthward. Thus, we see higher solar wind dynamic pressure during these years. Second, the lack of low IMF cone angles during these years is most likely due to the fact that dynamic pressure tends to be higher during high IMF cone angles than during low IMF cone angles (not shown). The numbers of magnetosheath intervals and jets closer to the model bow shock ultimately end up being small in those years, and these few intervals with a small number of jets have large weights in the yearly distributions.

**Figure 4:**
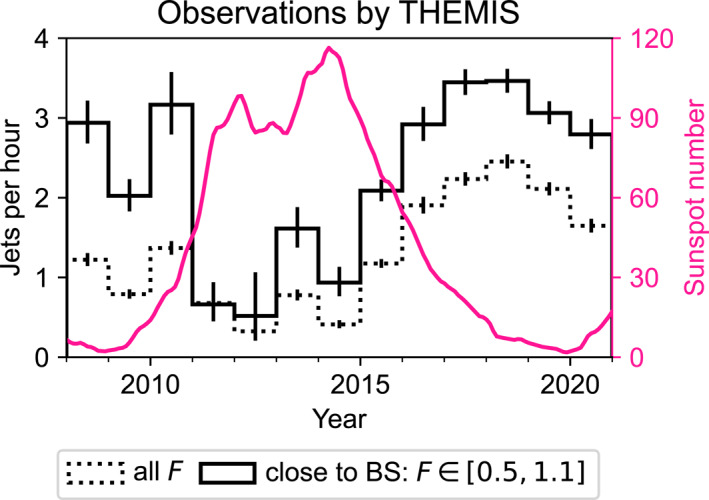
The yearly averages of observed jets per hour in the subsolar magnetosheath as observed by THEMIS spacecraft. The dotted histograms shows observations at all *F* values and the solid histogram shows the observations close to the bow shock (*F* ∈ [0.5, 1.1]). The error bars are 95% binomial proportional confidence intervals. The pink line shows the smoothed sunspot number from NOAA.

In Figure 4 we present the observed jet occurrence rates for all *F* values (dotted histogram) and only close to the bow shock (solid histogram). We have also overplotted the number of sunspots from NOAA (SILSO World Data Center, 1996–2021) as a function of time (pink line) to act as a measure of solar activity. There is a significant decrease in the jet occurrence rates in years 2011–2014, which leads to an apparent anti‐correlation: it seems as if jet occurrence is strongly decreased during the solar maximum. For all *F*, this decrease can be mostly attributed to the orbital differences. However, the jet occurrence close to the bow shock also seems to drop to ∼1 jet per hour from ∼3 jets per hour observed during other years. However, as shown in Figure 3, there is a bias in solar wind conditions during these years: the IMF cone angles during these THEMIS observations (Figure 3a) were notably higher than those expected by the OMNI observations (Figure 3h). Thus, these results in Figure 4 are not representative of the true jet occurrence rates.

## 3 Statistical Model of Jet Occurrence as a Function of Solar Wind Conditions

### 3.1 Method

**Figure 5:**
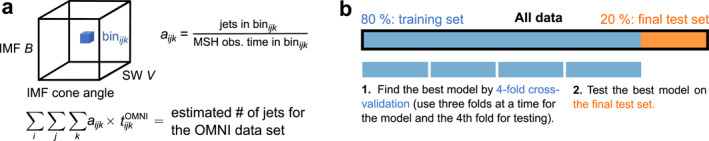
An illustration explaining (a) the “data cube” model, where we use existing data to estimate the occurrence rates of jets in each solar wind parameter space bin. New solar wind data can then be used as an input to the model (into the data cube) and the estimated number of jets for that data can be estimated; (b) the division of the THEMIS data set into a training set and a final test set, and how *K*‐fold cross‐validation (with *K* = 4) is used for model validation (i.e., for finding the best solar wind parameters for the model and the number of bins in the parameter space).

To obtain more representative estimates of the jet occurrence rates close to the bow shock during different years across the solar cycle, we create an empirical statistical model by using the THEMIS jet and magnetosheath observations close to the bow shock available to us, together with their OMNI conditions. Over the whole period of 2008–2020, THEMIS spacecraft have made an extensive number of measurements in the subsolar magnetosheath during different solar wind conditions. This allows us to construct a statistical model of jet occurrence as a function of solar wind parameters. For the reconstructions we use OMNI 1‐min resolution data, again averaged over the preceding 5 minutes. Using this extensive data set, we can calculate the number of jets seen per hour of magnetosheath observations during certain solar wind and IMF conditions (as illustrated in Figure 5a). For example, the statistical dependence of jet occurrence on the IMF cone angle is well known: jets occur mostly during low IMF cone angles, that is, downstream of the quasi‐parallel shock. We can use this statistical information to forecast/reconstruct how many jets per hour would we expect on average for given IMF cone angle conditions. We can also add other parameters to try to make the model better. We divide the parameter space into bins into which we project our jet and magnetosheath observations. We calculate the jets per hour occurrence rates *a*_*ijk*_ in all the bins (three indices *i*, *j*, and *k* would correspond to a model with three solar wind parameters). This “data cube” is our statistical model. To obtain an estimate from the model, we input OMNI solar wind data, again projecting it into the bins of the parameter space. Essentially the solar wind data set gives us a time $t_{\mathrm{ijk}}^{\text{OMNI}}$ spent in the conditions represented by bin_*ijk*_. We estimate that $a_{\mathrm{ijk}}\times t_{\mathrm{ijk}}^{\text{OMNI}}$ jets were seen during that time. To get the estimated total number of jets for a given set of solar wind data obtained over a period of time (e.g., a year), we simply sum over the number of jets obtained for all the bins. This is the method we are going to use in this paper to estimate the unbiased yearly jet occurrence rates.

This model is parametrized by the solar wind parameters used to create the data cube and by the number of bins in the parameter space. We select the model parameters from a pool of solar wind parameters which were found to influence jet occurrence based on the recent statistical results by LaMoury et al. (2021): IMF cone angle, IMF magnitude, flow speed, number density, plasma beta, and Alfvén Mach number. We divide each of the dimensions of the model parameter space into equal‐width bins either in linear or logarithmic space, depending on the parameter. This is done to best capture the influence of the solar wind parameter to jet occurrence. For the binning of parameters used in the models shown in this paper, we use (minimum, maximum, linear/logarithmic scale): IMF cone angle (0°, 90°, linear), IMF magnitude (10^0.06^ nT, 10^1.30^ nT, logarithmic), SW speed (280 km/s, 700 km/s, linear), and SW density (10^0.1^ cm^−3^, 10^1.4^ cm^−3^, logarithmic). We will search for the best model by using *K*‐fold cross‐validation (e.g., Hastie et al., 2009). The search is executed by systematically going through models with different solar wind parameter combinations and systematically increasing the number of bins in each of the dimensions. The best model is selected quantitatively by minimizing the maximum of our two error statistics, described below. Once the best model has been found during this *validation* step, we also test the final model's performance on new data quantitatively during the *final test* step.

To validate and test the model, we need to divide the data set into subsets. We do this by taking the individual intervals when THEMIS spacecraft were observing the subsolar magnetosheath and randomly assign these measurement intervals into subsets. We perform this partition separately for intervals of each year to ensure that all subsets have similar coverage over all years. As illustrated in Figure 5b, we use 80% of the data (the blue part) for validating and training the model and leave 20% of the data for final testing (the orange part). During *K*‐fold validation, we divide the training data into *K* = 4 folds, and each of the subsets (folds) is used once as a test set while the other three are used for training the statistical model. We evaluate the model with two error estimates. First, we assess its performance on a test set by comparing the yearly jet occurrence rates *a*_*y*_ (for year *y*) predicted by the model to the rates *b*_*y*_ actually measured in the test set for that year. We calculate the absolute error between these two values for each year and finally calculate a weighted mean of these yearly absolute errors (we weigh each of the yearly bins by the square root of the number of yearly magnetosheath observations *N*_*y*_ in the test set):

(2) $$E_{1}=\frac{\sum_{y}\sqrt{N_{y}}|a_{y}-b_{y}|}{\sum_{y}\sqrt{N_{y}}}.$$

Each validation cycle provides an error estimate (*E*_1,*n*_ for the *n*th cycle, *n* ∈ [1, *K*] = [1, 4]). We consider their average ${\bar{E}}_{1}=\frac{1}{K}\sum_{n=1}^{K}E_{1,n}$ to be the error of the model in the validation process. This first error estimate evaluates the predictive performance of the model.

Our second error estimate measures the stability of the model. Once we have created a model using training data, we have divided the parameter space into certain bins and calculated the jet occurrence rates *a*_*ijk*_ in each of those bins. We can also do the same thing using the test set—divide the test set data into the bins and calculate the jet occurrence rates *b*_*ijk*_ in them. This way we can measure how much the model (the jet occurrence rates in the bins of the parameter space) changes when the parameter space is filled by using different subsets of the data. We can again calculate the weighted mean of absolute errors between these rates (weighing by the number of all OMNI 2008–2020 observations $N_{\mathrm{ijk}}^{\text{OMNI}}$ in each bin):

(3) $$E_{2}=\frac{\sum_{i}\sum_{j}\sum_{k}N_{\mathrm{ijk}}^{\text{OMNI}}|a_{\mathrm{ijk}}-b_{\mathrm{ijk}}|}{\sum_{i}\sum_{j}\sum_{k}N_{\mathrm{ijk}}^{\text{OMNI}}}.$$

There will again be *K* = 4 errors each corresponding to one validation cycle (*E*_2,*n*_ for the *n*th cycle, *n* ∈ [1, *K*] = [1, 4]), and we average these errors to get an error estimate for the model that is used in the validation process: ${\bar{E}}_{2}=\frac{1}{K}\sum_{n=1}^{K}E_{2,n}$. This second error estimate ensures that our parameter space is not divided into too many bins (or dimensions) unnecessarily. Rather than choosing a marginally better model (in terms of predictive power) which includes many more bins, we favor a model with fewer bins as there is more statistical confidence in the rates of the bins. Weighing by all OMNI measurements from 2008 to 2020 ensures that the model performs the best during solar wind conditions that are the most prevalent (and have the most weight for the average yearly jet occurrence rates). We also tested weighing only by OMNI measurements from 2011 to 2015, that is around the time of the solar maximum where the jet occurrence rates observed by THEMIS were biased. The conclusions of this paper remained the same. We selected the largest feasible *K*, as we want to maximize the number of validation cycles. However, with increasing *K*, the sizes of the subsets become smaller and *E*_2_ becomes higher due to sampling error. *K* = 4 was found to be the best choice. We tested using *K* = 3 and *K* = 5, and the conclusions of this study remained.

During validation, we search for the type of model which minimizes the maximum of these two errors, $\max\left({\bar{E}}_{1},{\bar{E}}_{2}\right)$. Once we have chosen the best model (the best solar wind parameters and the best combination of the number of bins in the parameter space), we make the last test by using all the training data (80% of the data; blue part in Figure 5b) to train the model and test it on the final test set (the last 20% of the data; orange part in Figure 5b) that was left aside. Performing this final test on data that has not been used in creating the model allows us to test its performance on new data. We again calculate the error estimates *E*_1_ (Equation 2) and *E*_2_ (Equation 3) and consider max(*E*_1_, *E*_2_) as the final uncertainty of the model. After this we can start using the model: inputting OMNI data from the entire solar cycles 23 and 24 into the model.

### 3.2 Results of Validation and Testing

**Figure 6:**
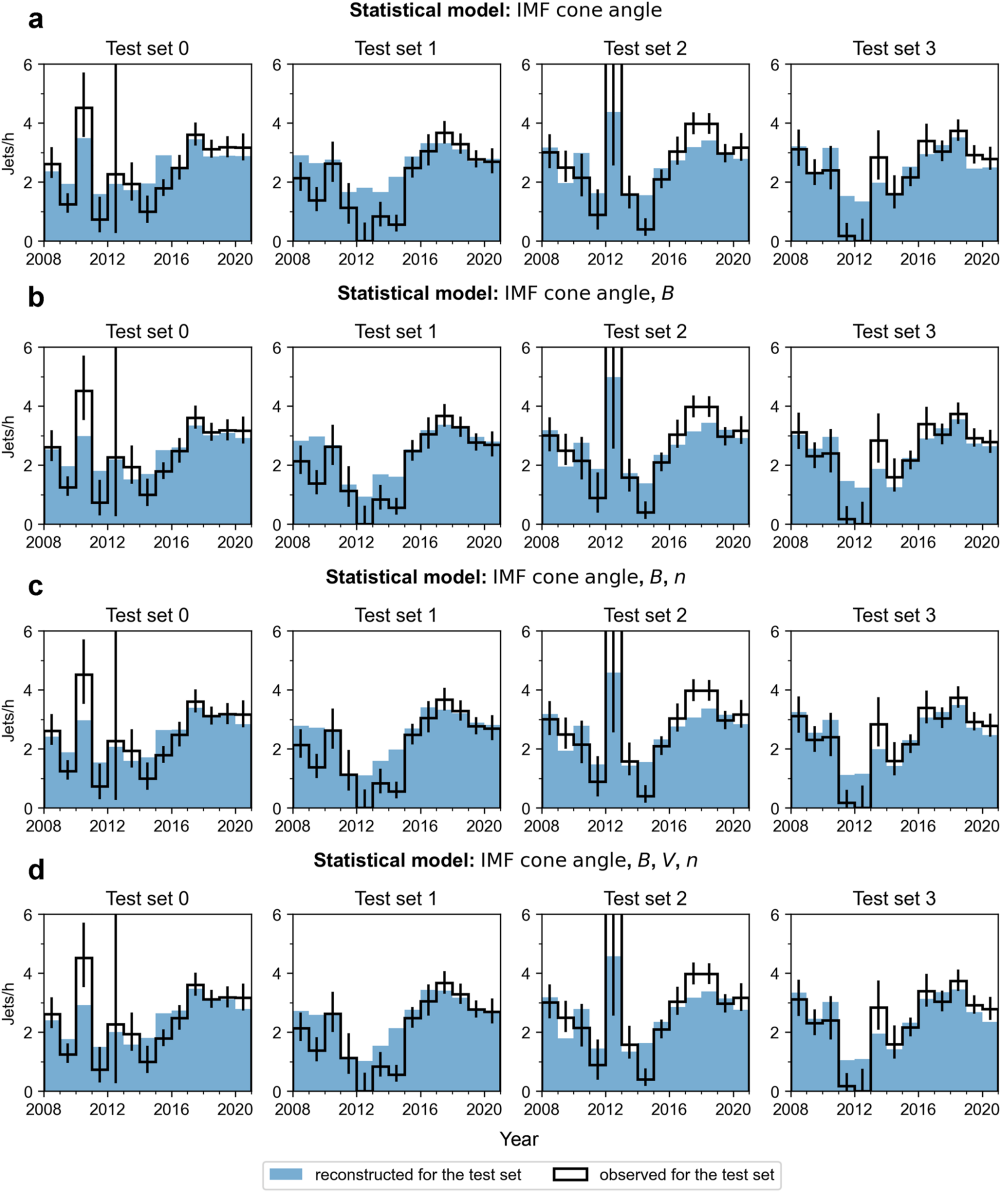
Results of the K‐fold cross‐validation, where the training data (80% of the THEMIS data set) has been divided into four folds. Each of the subsets is used once as a test set while the other four are used for creating the statistical models using (a) IMF cone angle, (b) IMF cone angle and magnitude, (c) IMF cone angle, IMF magnitude, and solar wind speed, and (d) IMF cone angle, IMF magnitude, solar wind speed, and density. The black histograms show the number of jets per observation time in the subsolar magnetosheath in the test sets. The error bars are 95% proportional confidence intervals. The blue histograms show the model predictions for the test sets.

In Figure 6, we show the results of 4‐fold cross‐validation for the best 1D model using IMF cone angle (with 16 linear bins), for the best 2D model using IMF cone angle and IMF magnitude (with 2 × 3 bins; linear, log), the best 3D model using IMF cone angle, IMF magnitude, and solar wind density (with 2 × 2 × 2 bins; linear, log, log), and the best 4D model using IMF cone angle, IMF magnitude, solar wind speed, and solar wind density (with 2 × 2 × 2 × 2 bins; linear, log, linear, log). The black histograms show the occurrence rates in the four test sets of THEMIS data. The blue histograms show the model reconstructions for these test sets. We note that especially during 2012 we can see large variations in the observed jet occurrence rates between the subsets due to the very low number of magnetosheath observations and jets during that year. The weighted mean absolute errors obtained for these models during the validation process are: 0.452 jets/hr, 0.399 jets/hr, 0.406 jets/hr, and 0.419 jets/hr, respectively. The 2D model with IMF cone angle and IMF magnitude is the best model. With *K* = 4, the parameter space errors *E*_2_ are more limiting than the yearly errors *E*_1_, because increasing the number of bins in the model often decreases *E*_1_ but increases *E*_2_. Thus, while some of the 3D and 4D models have slightly better yearly predictions, the uncertainty in the 2D model is lower. Furthermore, as those predictions of 3D and 4D models are only marginally better, this suggests that the parameters complementing the IMF cone angle and IMF magnitude are not so important. All of these four models seem to capture the yearly jet occurrence rates well, although not perfectly. There is enough predictive power to reproduce the dip during years 2011–2014, but the 1D IMF cone angle model does not reproduce it as well as the others. We note that have reproduced the final results of this paper also with these models that have lower *E*_1_, and the conclusions remain.

**Figure 7:**
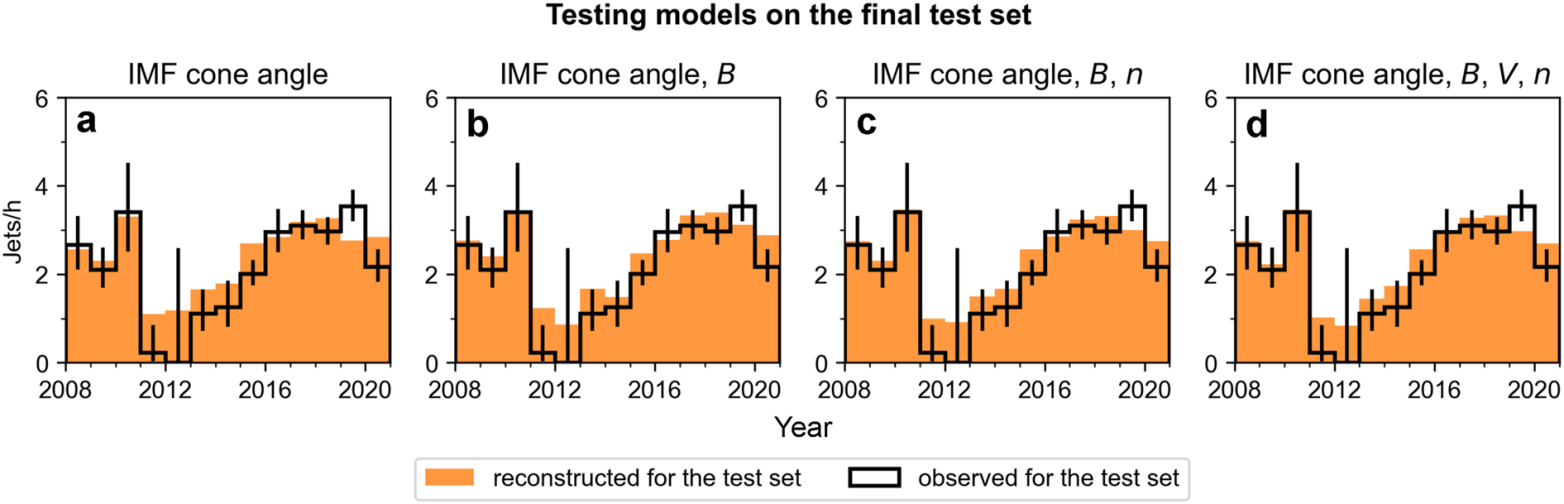
The results of testing the final models created using the whole training set (80% of the THEMIS data set) on the final test set (20% of the THEMIS data set). The orange histograms show the model predictions, and the black histogram shows the observed jet occurrence rates in the test set. The error bars are 95% binomial proportional confidence intervals.

Figure 7 shows the tests comparing the model predictions created using all training data to the final test set (20% of data that was reserved for this purpose). We again show the 1D (IMF cone angle), 2D (IMF cone angle and IMF magnitude), 3D (IMF cone angle, IMF magnitude and SW density), and 4D (IMF cone angle, IMF magnitude, SW speed and SW density) models. The final weighted mean absolute errors, that is, the uncertainties of the models, are 0.438 jets/hr, 0.386 jets/hr, 0.389 jets/hr, and 0.479 jets/hr, respectively. Again, the 2D, 3D, and 4D models capture the dip better. While the uncertainties are not negligible, they are, for example, much smaller than the dip in jet occurrence rates observed by THEMIS. Thus, the models will be accurate enough to determine whether there are strong variations in jet occurrence across a solar cycle.

### 3.3 Reconstructing Yearly Jet Occurrence Rates for Solar Cycles 23–24

**Figure 8:**
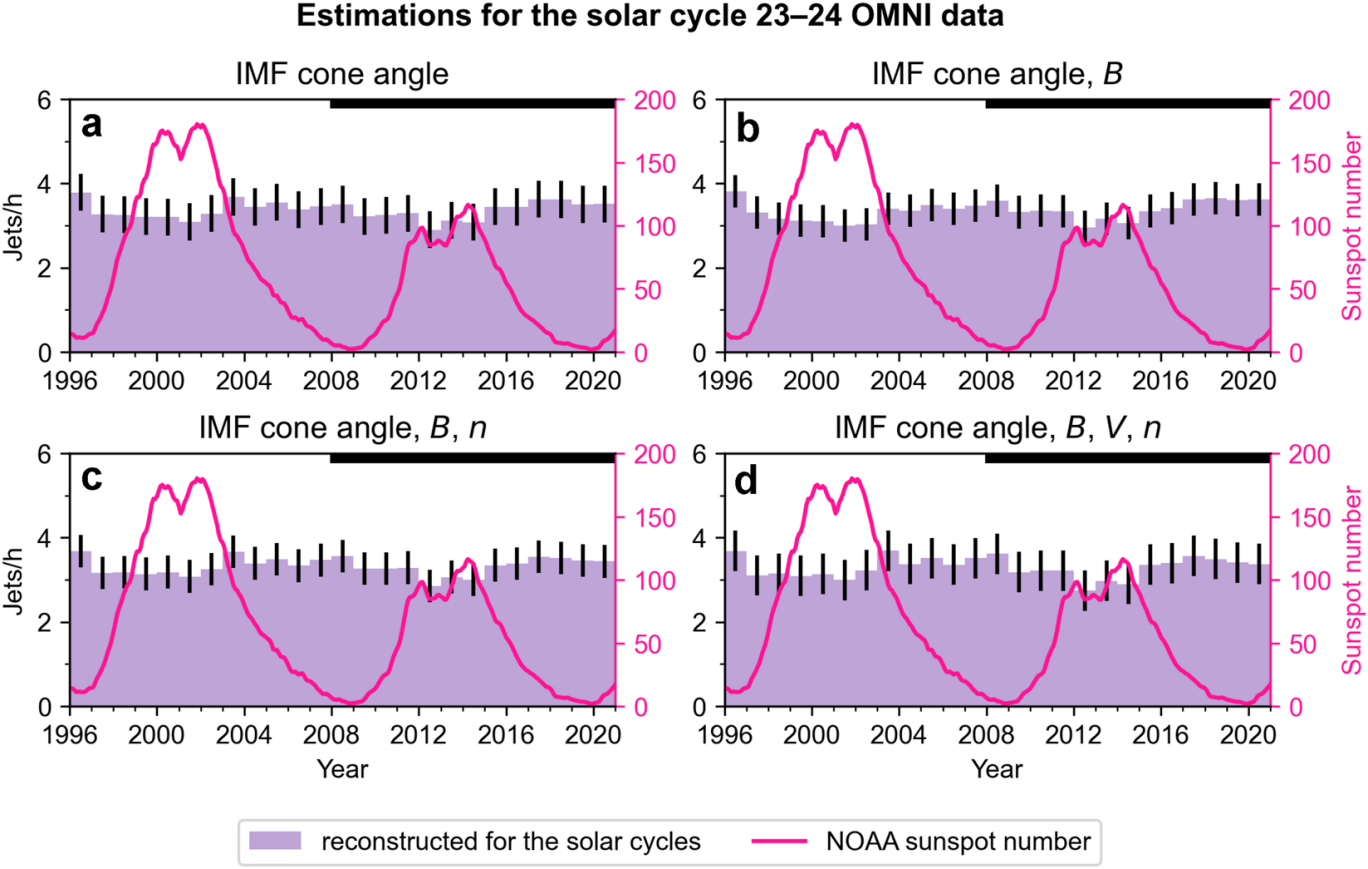
Results of the statistical model (using IMF cone angle, IMF magnitude, solar wind speed, and density) applied to the OMNI data of years 1996–2020 (spanning over solar cycles 23 and 24). The error bars denote the estimated uncertainties of the models. The pink line shows the smoothed sunspot number from NOAA. The thick black horizontal lines at the top of the panels highlight the years 2008–2020, to which we can compare the results of Figure 4.

Finally, we input the entire OMNI solar wind data of the solar cycle 24 and estimate the yearly jet occurrence rates. The OMNI data is in the same format as used in the statistical data set when building the model: 1‐min resolution data averaged over the five preceding minutes. To understand the trends more generally, we also model the solar cycle 23. We show the reconstructed jet occurrence rates per year obtained from the four different models in Figure 8 (the purple histograms), using the model uncertainties as error bars. The models produce almost identical results, which indicates that the IMF cone angle is enough to capture the statistically most important solar wind variations influencing jet occurrence. We have again overplotted the sunspot number as a function of time. The solar cycle 23 was a more active cycle than cycle 24, as clearly evidenced by the significantly higher number of sunspots. We can see that there is no strong decrease in jet occurrence during the solar maximum of solar cycle 24 that would correspond to the dip seen in the THEMIS observations in Figure 4 (see the solid black histogram, which we concluded was not representative of the annual solar wind distributions). The histograms show a shallow (around 10%–20%) dip at the maxima of both solar cycles, but they are within the uncertainties of the model. Thus, our model results indicate that there is no strong solar cycle variation in jet occurrence.

## 4 Discussion

We have used THEMIS observations from the subsolar magnetosheath spanning years 2008–2020 to study how jet occurrence varies throughout the solar cycle 24. However, we find that the average yearly occurrence rates are not directly comparable to each other, complicating this investigation. The THEMIS spacecraft apogees changed throughout the years, and the spacecraft spent relatively less time close to the bow shock when the apogees were lower, especially during the years 2010–2014. This affected the number of observed jets during those years, because jets are more common near the bow shock. Therefore, such an effect should be accounted for when aiming for the unbiased jet occurrence rates. We have considered this by only using data close to the model bow shock. However, when the spacecraft apogees are lower than usual, this selection favors solar wind conditions during which the magnetosphere is compressed and the bow shock moves earthward, that is, times of high solar wind dynamic pressure. Therefore, we find that the distribution of solar wind conditions during the THEMIS measurement intervals close to the bow shock is not representative of the true distribution of solar wind conditions as observed by OMNI during those years. Additionally, THEMIS spacecraft traverse the subsolar magnetosheath for only a fraction of a year. The solar wind properties vary within a year, and thus the distribution of solar wind conditions during a THEMIS observation interval may differ from the distribution throughout the entire year.

To account for the orbital bias and bias due to uneven solar wind sampling in the measurements of different years, we have created a statistical model of jet occurrence close to the bow shock as a function of solar wind conditions. We have used the THEMIS observations from 2008 to 2020 to create the model. This model allows us to input unbiased OMNI solar wind observations throughout the entire solar cycles 23 and 24, and to estimate less biased average yearly jet occurrence rates. According to our model, jet occurrence does not vary strongly within the solar cycle. There might be a slight (around 10%–20%) decrease in jet occurrence during solar maximum which is, however, within the uncertainty of the model. This decrease was observed in all the presented models in this paper and for both the solar cycle 23 and 24. The best model with the lowest error estimate used two parameters: IMF cone angle and IMF magnitude. The predictions for higher‐order models with solar wind speed and density are slightly better, but the sampling errors (Equation 3) increase, making these models less reliable. The model that used only IMF cone angle also produced very similar predictions for the solar cycles 23 and 24. This suggests that variations in IMF cone angle are the dominating component in variations of the absolute number of jets. This is understandable because jet occurrence rates are 9 times higher during low (<30°) IMF cone angles than during high (≥60°) IMF cone angles (Vuorinen et al., 2019).

OMNI high‐resolution data set contains data combined from multiple spacecraft. Over the years 1996–2020 investigated here, the data set contained observations from: ACE (1998–2020), Geotail (2001), IMP‐8 (1996–2000), and WIND (1996–2020). While ACE measurements (McComas et al., 1998; Smith et al., 1998) comprised most of the OMNI data for solar cycle 23, WIND observations (Lepping et al., 1995; Ogilvie et al., 1995) dominate the data set for solar cycle 24. It is important to point out that the OMNI data set has a better coverage for magnetic field data than for plasma data. The yearly magnetic field data coverage varied between 85% and 96% (mean 92%) during the years 2008–2020. The yearly coverages for plasma data varied between 69% and 85% (mean 78%). Therefore, the model that only uses magnetic field data may be preferred. We note that these percentages of OMNI coverage also apply to the data that we have input to our model in this study. However, these two‐parameter and the three/four‐parameter models produced very similar results, which indicates that this is not an issue. Overall the proportions of the year when OMNI data was not available at all varied between 4% and 15% (mean 8%), with 2014 having clearly the worst coverage. All in all, because this was typically a small fraction of the data and our solar wind parameter bin size is coarse, we do not expect it to be significant for the results.

We note that the annual jet occurrence rates are not an estimate of the number of all jets that occurred in the magnetosheath close to the bow shock, but rather an estimate of how many jets per hour a THEMIS spacecraft would have observed if it was observing jets close to the bow shock continuously. The THEMIS spacecraft cannot observe all jets, but how their observed jet occurrence rates change allow us to estimate how the total jet occurrence rates vary. As mentioned in Section 2.1, we repeated the analysis neglecting THEMIS E data, to ensure that THEMIS E on‐board moment calibration issues do not change the results. The reconstructed average yearly jet occurrence rates decrease by 25% (from rates ∼3–4 jets/hour to ∼2–3 jets/hour), but the trends and our main conclusion remain: there is no strong solar cycle variation in jet occurrence.

Koller et al. (2022) studied the connection between jets and large‐scale solar wind structures, and found that the occurrence of jets (defined by Plaschke et al., 2013, criteria) increases by ∼20%–50% during SIRs/CIRs and HSSs, but decreases by ∼0%–30% during sheath regions of CMEs and by ∼20%–60% during their magnetic ejecta. CMEs are most frequent during solar maximum when flows related to them can constitute up to ∼40%–60% of the solar wind at Earth (e.g., I. G. Richardson & Cane, 2012). SIRs or CIRs are more frequent during the declining phase of the cycle when they can make up around 60% or more of the solar wind flow at Earth (e.g., I. G. Richardson & Cane, 2012). While our results indicate that there are no strong statistical variations in the average yearly jet occurrence rates, solar wind structures and periodic variations in the solar wind can still modulate jet occurrence. More studies are needed to understand the ranges of jet occurrence rates during different types of events. Here we have focused on jet formation, but jet propagation to the magnetopause is enhanced during high solar wind speed (LaMoury et al., 2021). This means that solar cycle periods with higher solar wind speeds may lead to more geoeffective jets.

## 5 Conclusions and Summary

Yearly THEMIS observations of jet occurrence rates are biased due to variations in spacecraft apogees in the subsolar magnetosheath and uneven coverage of the yearly solar wind conditions. Considering these biases in the data is crucial, because improper normalization can affect the conclusions drawn from the observations. This issue is not unique to jets, but also concerns other phenomena that are dependent on solar wind conditions and/or position in the magnetosheath.

Leveraging the information contained in the vast amount of THEMIS data, we have created an empirical statistical model of magnetosheath jet occurrence as a function of solar wind conditions and used it to reconstruct unbiased estimations of yearly jet occurrence rates across solar cycles 23 and 24. The best model (that minimizes the error estimates) has two parameters: IMF cone angle and IMF magnitude. 3D and 4D models with solar wind speed and density also included can provide slightly better yearly predictions, but the statistical errors become larger due to the finite size of the data set. Even a 1D model with just the IMF cone angle produces similar results and identical conclusions. Our model results show that the occurrence rate of earthward magnetosheath jets close to the bow shock does not vary significantly across the solar cycle. Both solar cycles exhibit a decrease of the order of 10%–20% near the solar maximum, but this is within the uncertainties of the model. In the future, the statistical model can be further improved by including more data, either measurements from other spacecraft missions or future THEMIS observations.

## Data Availability

THEMIS and OMNI data can be accessed via, for example, NASA's Coordinated Data Analysis Web (https://cdaweb.gsfc.nasa.gov/). The magnetosheath and jet data set used in this study can be found at Koller et al. (2021). The empirical models used to produce the final results of this study can be found at Vuorinen et al. (2023).
